# Electrolysis, and Its Application to the Treatment of Disease*Read before the New York Medical Journal Association, February, 1871.

**Published:** 1871

**Authors:** A. D. Rockwell

**Affiliations:** New York


Art. I.—*Electrolysis,* and its Application to the Treatment of Disease.* By A. D. Rockwell, M.D., New York.

The increasing interest manifested by the profession in all the departments of electro-therapeutics and electro-surgery induces me to offer a few words on electrolysis—a subject of the greatest importance, and, in its application to disease, one of the most fascinating studies to which the human mind can be brought. Before proceeding to discuss the action of the galvanic current on the dead and living tissue, I will define some of the terms that are commonly used.

An electrolyte is any compound substance that can be directly decomposed by the current.

To electrolyze a body is to chemically decompose it by the current. Substances widely vary in the readiness with which they are decomposed, but manifestly no substance can be an electrolyte unless it is in some measure a conductor. Every electrolyte must contain more or less of water; but pure water is decomposed with difficulty, and the readiness with which any electrolyte is decomposed depends on the quantity of certain salts contained in solution. Hence, blood and muscular tissue are good electrolytes.

The anode and cathode refer to the points of the decomposing body that are in contact respectively with the positive and negative pole. Electrolytes are resolved under the action of the current into anions and cations, which appear at their respective electrodes in the proportion of their atomic weights or multiples of their atomic weights. Water, for example, is an electrolyte

*Read before the New York Medical Journal Association, February, 1871.

that evolves two *ions*—oxygen and hydrogen; oxygen goes to the anode, and is the anion; hydrogen goes to the cathode, and is the cation.

| Electrolytes. | Composition. | Cations. | Anions. | Relative Proportions. |
|---|---|---|---|---|
| Hydrochloric acid, | H.CL. | H. | CL. | 1 to 35.5 |
| Chloride of Sodium, | Na Cl. | Na. | Cl. | 23 " 35.5 |
| Sulphuric acid, | $H_2SO_4$ | $H_2$ | $SO_4$ | 2 " 96 |
| Sulphate of soda, | $Na_2SO_4$ | $Na_2$ | $SO_4$ | 46 " 96 |

The galvanic or constant current is chiefly used to obtain electrolytic action, although the faradic current undoubtedly possesses some chemical power.

In regard to the conditions of quantity and intensity, it must be remembered that, in the operation of electrolysis, they are exactly the reverse of those required for the purposes of galvano-cautery. For the latter operation, quantity with moderate tension is required, and this is obtained by a considerable number of elements of medium size.

The statement that very large elements are necessary, or that only some special form of battery will produce results, is undemonstrable. Some batteries are more convenient than others, and give a more constant current, but all will, to a greater or less extent, produce decomposition. If the current be weak, then the applications may be longer. It is doubtless better, however, to use elements of good size. An evidence that cells that are very small and that give comparatively little quantity, will yet produce electrolysis, is found in the fact that decomposition of inorganic substances in water is effected, as Faraday and others have shown, by statical electricity and the voltaic pile. Now, the quantity of statical electricity is exceedingly small, and the quantity of the voltaic pile is not great; therefore, it follows that a very large quantity is not necessary to produce electrolysis, and small, even very small, elements will decompose.

For the purpose of electrolysis, when the action of the positive pole is desired, the needles should be of some metal that readily resists oxidation. Gold or gilded steel needles answer well for the negative pole. The needles should be insulated at the upper part, with hard rubber, to prevent the action of the current on the skin and subsequent inflammation.

The electro-chemical action that takes place during the operation of electrolysis is by no means thoroughly understood. The researches of Faraday, however, have demonstrated that where several substances are decomposed by the action of the current, the elements evolved are definite in quantity and are electro-chemical equivalents of each other. It has been found, also that electrolytic action is very materially modified according to the nature of three important factors, viz: 1. The composition of the substance decomposed; 2. The material of which the electrode is made; 3. The strength of current employed. The iodide of potassium, for example, readily decomposes under a very feeble current—the iodine and oxygen going to the positive, while the hydrogen and alkalies go to the negative pole, changing the solution to the color of iodine. If, now, we substitute for the platinum electrode connected with the positive pole an electrode of lead, the lead immediately oxidizes, and combining with the iodine, deposits the iodide of lead. A solution of common salt, also, when submitted to the influence of the galvanic current by means of platinum electrodes, decomposes almost as readily as the iodide of potassium. Chlorine appears at the positive, and hydrogen and oxide of sodium at the negative pole. By subtsituting, again, for the positive platinum electrode an electrode of copper, the copper oxidizes, combines with the chlorine, and forms chloride of copper. These simple tests well illustrate the important modifications produced during electrolytic action, according to the nature of the electrodes employed.

For electrolytic experiments on various substances, platinum is the best electrode, because it is not acted on. Copper and other metals may be used, but the secondary action which occurs in the decomposing body greatly complicates the experiment.

Electrolysis is produced most quickly and effectually when the current is made very dense by the use of small needles ; it is, however, entirely demonstrable, that definite electro-chemical action is excited when either one or both of the electrodes are broad, flat disks. By substituting for the needle or wire electrode in the potassium solution a flat disk, decomposition at once takes place, and if the strength of the current is sufficiently increased to compensate for the decrease of density by the increase in the size of the electrodes, the solution will almost immediately begin to assume the color of iodine. From this illustration it can be readily conceived that electrolytic as well as electro-tonic effects may be produced by the ordinary applications of galvanism, and especially when ulcers are treated by metalic plates or moist sponges.

In addition to the solutions already mentioned, we have experimented on a variety of fruits and vegetables, and also upon eggs, blood, and dead tissue. In most fruits and vegetables electrolytic changes take place with readiness, on account of the large quantity of water and acids they contain. When a pear, potato, or an apple, is submitted to electrolyzation, the part pierced by the negative pole changes in color, and withers. The process of drying and discoloration goes on after the operation is discontinued. The phenomena that occur during electrolyzation of the white of an egg are very striking. White flakes form rapidly around the negative pole. These soon become detached, and float upon the surface of the albumen. The process may be repeated until the whole surface is covered with a white mass, resembling froth.

This mass is not coagula, but is formed by the nascent hydrogen, mechanically driving asunder the particles of albumen.

The phenomena that are presented during electrolysis of dead tissue afford an interesting and suggestive study. During the passage of a galvanic current by means of needles through a piece of raw beefsteak, bubbles of hydrogen appear at the negative pole, while the tissue in the immediate neighborhood becomes decidedly shrunken. At the positive pole a white froth appears, chlorine is evolved through decomposition of the saline solutions, and the part surrounding the needle becomes hard and tough. This process of decomposition is attended by a very distinct hissing or frying sound, and the temperature of the part that is being electrolyzed is decidedly increased. Chemical examination shows that oxygen and albumen go to the positive, while hydrogen and coloring matter go to the negative pole. The beef gradually becomes dry and changed in color, its watery constituents are absorbed, and so electrolytic action becomes less and less decided until it altogether ceases. For some hours subsequent to this process, that portion of the beef acted upon continues to contract and shrivel up, until it presents the appearance of a charred mass.

When fresh blood is submitted to the action of the galvanic current, foam of a light-yellowish color collects on and around the negative pole, while near the positive a dark, clotty mass appears, in strong contrast with the color of the surrounding blood. As might be supposed, litmus-paper gives at the anode an acid reaction, and at the cathode an alkaline reaction.

Dr. Victor V. Bruns,* in his late work on galvano-caustic and electrolysis, has recorded some interesting experiments on the circulating blood. His method of procedure was to open an artery or vein, and by judicious and well-directed pressure to prevent both the escape of the fluid and its two rapid flow. He then inserted into the opening a needle connected with the positive pole, when a light-colored, spongy clot rapidly formed around it and adhered also to the coat of the artery. At the negative pole, when applied in the artery, no clot was formed, but an abundance of gas and bloody foam appeared.

The relations which the very interesting and suggestive phenomena here recorded sustain to the practical application of electrolysis in the treatment of disease, are not sufficiently understood to render the subject a complete or exact science.

Clinical experience is the only sure basis on which to build, and this teaches that living is more readily electrolyzed than dead tissue. This is accounted for from the fact that living tissue is capable of the process of absorption, and that its solutions are warmer and therefore better conductors. When, therefore, a

*Die Galvano-Chirurgie oder die Galvano-Kaustic und Elektrolysis bei chirurgischen Krankheiten. Tubingen, 1870; p. 73.

tumor capable of being electrolyzed is submitted to the action of the galvanic current, a threefold action is produced:

1. Its fluid constituents suffer decomposition. Hydrogen and alkalies, soda and potassa, go to the cathode, and oxygen and acids to the anode. While electrolytic action thus takes place at both poles, it is evident that this action is most vigorous and more readily produces absorption in living tissue at the cathode. At the anode, however, decided chemical action takes place and successful results are obtained by it. But since, as we have demonstrated, electrolytic action is modified by the composition of the electrolyte, and the character of the poles, it is probable that a more extended clinical experience will establish more definitely the important fact that some conditions of disease are most successfully treated by the positive, and others by the negative pole.

2. Absorption is hastened by the chemical effects of the current and the mechanical and irritating effects of the needles, and may slowly continue for weeks.

3. Disintegration and atrophy take place. If the part acted on by the current be a small wen, wart or nævus, the tissue may become changed in color, dried and shrivelled, and almost entirely disappear during the operation.

A word concerning the general method of electrolyzation, and the method of testing batteries to ascertain their comparative advantages for electrolysis. In treating the various forms of tumors, aneurisms, and varicose veins, serous effusions, wounds and ulcers, both poles may be made to operate simultaneously; or, if only the negative pole is used, the current is completed by placing the positive, connected with a sponge electrode, on a neighboring part.

Considerable pain is caused by the introduction of the needles, and frequently an anæsthetic is necessary. The length of the *seance* varies from a few minutes even to one hour. In treating tumors, much depends upon their character. The intensity of the current and the length of the *seance* depend, as has been shown, on the special purpose in hand. The general rule is, the harder and larger the extent of tissue to be acted on, the greater the quantity and intensity of electricity required. The composition of an erectile tumor is such that it offers but little resistance to the passage of the current and readily electrolyzes, while a scirrhous tumor offers great resistance, is with difficulty acted upon, and during and immediately after electrolysis may exhibit no change, except a slight enlargement and softening due to the disengagement of its gases.

Through the researches of Faraday we can test the quantity of electricity generated by any form of battery, by means of a galvanometer. But, in the operation of electrolysis, intensity also is needed, and a rough but approximate test for both quantity and intensity is found in the iodide of potassium. The rapidity with which this yields to the current, and the amount of iodine evolved in a given time, or quantity of water decomposed, approximately indicate the capacity of a battery for electrolytic purposes.

When we come to inquire concerning the practical results yielded by electrolyzation in its application to tumors and other surgical diseases, the field opened before us is a most encouraging one. A great variety of abnormal growths, nævi and papillary enlargements, sebaceous, hydatid, and erectile tumors, goitres, and even cancers, are reported as having been successfully treated by electrolysis. Our own experience in this department of electro-therapeutics, not yet very extensive, has been on the whole measurably successful.

### MORBID GROWTHS (NON-MALIGNANT).

Case I.—The first was an erectile tumor of small size, located on the penis. A needle connected with the positive pole was introduced into the *mass*, and a current from ten cells of Storber's battery was allowed to pass for exactly five minutes. During the operation the tumor could be seen to change in color, and to rapidly decrease in size. At the close of the *seance*, hardly a trace of any enlargement could be felt.

Case II.—In April of this year, I saw, with Dr. D. F. Reynolds, a little child aged eight months, afflicted with an erectile tumor in the right cheek, that measured about one and a half inches in width, and from one-half to three-quarters of an inch in depth. In the presence of Dr. Frank Hamilton and his class, I operated at Bellevue Hospital, by introducing into the four quarters of the tumor four small gilded-steel needles, insulated to within one-quarter of an inch of the points. During the passage of a current of very moderate tension, the enlargement gradually grew harder and more prominent as the blood coagulated, and at the expiration of eight minutes, when the needles were withdrawn, the part was quite solid. The process of absorption soon became manifest, and at the present date, June 12th, it is but one-fourth ($\frac{1}{4}$) of its original size. Absorption still goes on, and it will take but a few more weeks for every vestige of the tumor to disappear.

Case III.—A patient, sent to us by Dr. Andrews, to be treated for a form of paralysis, complained also of a soft tumor about the size of a walnut, and situated on the back near the spine. The enlargement appeared to be fatty in character. Needles connected with both the positive and negative poles were introduced into the tumor, and a current of great tension from eighteen cells was allowed to pass for twenty minutes. During the *seance* the tumor changed in color and grew smaller, and in a few days subsequently disappeared entirely.

Bruns* reports a remarkable case of fibrous polypus of the naso-pharyngeal space—that had been once removed by a surgical operation, but which very soon returned, and became so large

* Op. cit., p. 85.

that it projected into the mouth, and also into the left nasal passage. The electrolytic treatment was begun May, 1869. One needle was put into the naso-pharyngeal, and the other into the nasal portion. The patient was treated in this way, with some short interruptions, over ten months. By March, 1870, one hundred and thirty sittings had been held.

The polypus was so much reduced in size, that it could not be seen either through the nasal passages or in the mouth, and only a small portion remained that could be felt by the finger. Improvement began soon after the electrolytic treatment was commenced.

Case IV. *Fibrous Tumor of the Uterus.*—I have had under observation, a case of fibrous tumor of the uterus, and although the electrolytic process, by means of the introduction of needles, has not been employed, it may be proper to refer to it in this connection.

The patient is under the care of Dr. Peaslee, who desired me to test the effects of some form of electrolyzation. The tumor is sub-peritoneal in character, on the right side, and about the size of the two fists. The resultant symptoms of which the patient especially complained, were severe shooting-pains down the right leg, together with a decided anæsthesia, and loss of power that seriously interfered with locomotion. I alternately passed both currents through the tumor, using the faradic internally and the galvanic externally.

A few applications entirely dissipated the severe pain and the anæsthesia, and restored the strength of the limb.

It is not impossible that this treatment, persevered with faithfully, may, in time, cause a reduction in the size of the growth. Should it fail to do so, it may possibly be advisable to resort to electrolyzation, by introducing insulated needles into the mass.

**Cancers.**—In regard to the electrolytic treatment of cancers, M. Bruns* remarks that in no case has he obtained a complete removal of, or even a very great reduction in the size of, such tumors, by the absorption resulting from the electrolytic process. He states that the parts of the tumor where the needles are inserted become more or less burned or destroyed, and that suppuration takes place, afterward granulation and cicatrization, and finally some shrinking, with a slight reduction in the size of the tumor. If a number of needles were inserted so as to cut off some part of the tumor, the part so enclosed could be destroyed. In this way he succeeded in reducing the size of a number of cases, but complete atrophy or absorption, as a result of this treatment, he has never seen. He states that, perhaps, his ill-success may be due to the fact that the patients did not persever.

Gherini and Althaus, who have studied this subject, have

*Ibid.

obtained only incomplete results—a diminution of the lancinating pains, but no complete removal. Manfredieu (1869), reports a complete removal of a "cancer villosus of the lower part of the thigh by electrolysis."

One successful and a number of unsuccessful attempts in the treatment of this disease we have reported elsewhere.

An operation that was performed in one of the wards of Bellevue, in the presence of Profs. Wood, Hamilton and other physicians, failed to accomplish successful results. It was satisfactory in so far as it seemed to show that, in a certain class of morbid growths of a semi-malignant character, electrolytic action may have a tendency to accelerate rather than retard progress.

Case V.—The patient, a robust young man, of twenty-five or thirty, was suffering from a myxosarcoma as large as the two fists, situated on the left pectoral muscle. The tumor had already been removed by Prof. Wood, and at his suggestion it was decided that electrolysis should be used. I operated by the needles on three different occasions, at intervals of about a week. After each operation a large quantity of bloody foam, formed by the action of the nascent hydrogen, escaped from the parts penetrated by the needles connected with the negative pole. The tumor became decidedly softer; but in this case the irritation caused by the current evidently tended to hasten ulceeration, and to produce unsightly fungoid growths around the holes made by the needles. The tumor was subsequently removed a second time by Prof. Wood.

Ulcers.—The continuous current applied to chronic ulcers will frequently produce a growth of healthy granulation after all ordinary means have failed.

Case VI.—The rapidity and completeness with which the electrotonic and electrolytic effects of the current may occasionally be manifest, in the healing of ulcers, were observed a short time since in the case of a little patient of mine. An abrasion near the ankle—some twelve months before—had resulted in an ulcerated surface about the size of a silver dollar. The ulcer was in a relaxed, torpid condition, and had resisted the ordinary applications. I made an application of the faradic current around the outer border of the diseased part, and then with the positive electrode of silver placed over the ulcerated surface, and the moist sponge of the negative pole applied to the opposite portion of the limb, a mild galvanic current from four zinc carbon cells was allowed to pass for five minutes. On the following day the operation was repeated.

I saw no more of my patient until several months subsequently, when the mother called at the office with the child. She stated that, a few hours after the second operation, a fine gauze-like covering formed over the ulcer, gradually growing thicker, until a firm scab covered the part. In about two weeks the scab fell off, leaving exposed a healthy surface, which quickly healed.

**Skin-Diseases.**—A number of the diseases of the skin may be treated electrolytically by means of broad electrodes of various shapes and sizes, covered with flannel, linen, or with simple metalic plates. It is probable that every passage of a galvanic current of much strength through the body is attended by more or less electrolytic action, and it may with entire reasonableness be presumed that, in all ordinary applications of the galvanic current to the central or peripheral nervous system, a tendency to chemical change is excited.

Case VII. *Case of Eczema.*—In the case of an old gentleman, who had for some months suffered from the chronic form of eczema of the face, the electrolytic and electrotonic effects of the current were strikingly manifest. Alternate applications to the diseased part, of the galvanic and faradic currents, were followed by a favorable change in the character of the eruption in less than a week, and at the present date, less than a month since the first application, the cure is approximately accomplished.

Case VIII. *Case of Psoriasis of Twenty-two Years' Standing.**—A case of psoriasis of twenty-two year's standing, with large spots on the hypochondriac and epigastric organs, and on the legs, in a woman of middle life, was treated by galvanization of the spots, together with galvanization of the smpathetic. Both poles were used, but chiefly the negative. The effects were decided and immediate, and in the course of two months the spots had disappeared. The treatment was given three or four times a week. To differentiate between the effects of the peripheral and central treatment was hardly possible. The question whether galvanization of the spine or sympathetic may of itself have an effect on psoriasis, or other diseases of the skin, can only be settled by a number of comparative observations.

In another case of psoriasis, peripheral galvanization in a man of middle life has obtained decided though not rapid results, and it is now proposed to combine, with the peripheral treatment, central galvanization.

Casa IX. *Sclerosis of the Skin.*—A case of Sclerosis of the Skin, a disease which is usually regarded by dermatologists as incurable, has derived benefit from galvanization, chiefly with the negative pole, and with a current of as much strength as could be conveniently borne. The patient is still under treatment.

Case X. *Prurigo.*—A case of Prurigo in a young man was treated by sponge electrodes over the whole surface. Benefit at once appeared. The itching diminished, and the appearance of the disease improved. In a short time, and by a few applications, the recovery appeared to be complete.

In all these cases the electrolytic effects of the galvanic current unquestionably had much, if not nearly all, to do with the results that were obtained; although the current may, as has been

---

* The three following cases were treated, in conjunction with Drs. Piffard and Beard, at the Dispensary for Skin-Diseases.

remarked, have other effects which, perhaps, we may never fully understand.

Certain it is that a strong alterative action takes place when either sponge or metalic surfaces—connected either with the positive or negative pole of a galvanic current—are applied to the diseased skin. It may be remarked that some diseases of the skin, as prurigo, may also be treated by the faradic current, which possesses little if any electrolytic power.

It is yet too early to express absolute opinions concerning the electro-therapeutics of dermatology, since the first and so far as we know, the only systematic experiments in this department are those here recorded; but it is safe to predict that the future is full of promise, and that electricity, especially the galvanic current, will prove an undoubted adjuvant in the treatment of very many of the obstinate and hitherto called incurable diseases of the skin.

**Stricture of the Urethra.**—Strictures of the Urethra have been treated electrolytically by Mellez and Tripier.* They report forty cases. Their method was to introduce to the stricture a metalic bougie, insulated to within a short distance from the extremity. This was connected with the negative pole of a galvanic current of from five to fifteen or twenty elements, while the positive pole, by means of a moist sponge, was applied somewhere on the thigh. The sittings lasted from five to fifteen minutes. Improvement was often immediate, and frequently a bougie would pass easily, which before could not be introduced. The pain of the operation was slight, a little blood appearing on the withdrawal of the bougie. The first urination was easy and painless, but subsequently some pain would be experienced on urination. After five or six days, the cauterized tissue would be thrown off. The cures were permanent. Febrile symptoms occurred only in exceptional instances, and were only light and short. It should be stated, however, that one patient died.

Couriard, of St. Petersburg, has treated twelve cases by this method, in three of which abscesses appeared in the surrounding tissue. In one case the application was continued for forty minutes, with the effect of widening the urethra, but without opening the stricture, so that the bougie could be passed. In ten cases there was more or less improvement. Couriard, though he was not so successful as Mallez and Tripier, yet recommends the treatment for those cases that do not yield to dilatation, and as preferable to urethrotomy.

**Other Diseases for which Electrolysis may be used.**—Among other conditions for which electrolysis has been used with varying success, are : aneurisms, varicose veins, ascites,

---

* De la guerison durable des retrecissements de l'urethre par la galvano-caustique chimique, par F. Mallez et A. Tripier. Paris, 1867. Translated and condensed in this Journal for February, 1868, by Dr. R. D. Nesmith.

hydrocele, pleuritic effusions, goitre, urethritis, nævus, rhinitis, and ozæna. The scope of this essay will not permit a detailed consideration of these diseases. Among many decided failures in the treatment of aneurisms and varicose veins by electrolysis, some absolute successes have been reported.

The method of procedure is, to introduce into the diseased vessel one or more needles, connected with one of the poles, while the other pole may consist either of a moist sponge, placed on a neighboring part, or of needles penetrating the artery in close proximity to, but not touching, the needle of the pole first introduced. It has been asserted that successful results have been obtained by the action of the negative pole alone. It is claimed by Althaus that at the cathode a slow but firm deposition of fibrine takes place, that gradually but surely strengthens the walls of the aneurism, while at the anode a clot is rapidly formed, but is loosely attached to the coat, and may readily be washed away. An experiment by Drs. Keyes and Beard on the abdominal aorta of a dog, did not seem to confirm this statement. While a decided clot was formed at the positive pole, there was no appreciable deposition of fibrine at the negative pole.

Certain forms of serous effusions, especially hydrocele, have been treated with marked success by galvano-puncture. The hydrogen and alkalies that are disengaged at the negative pole, exercise both a mechanical and chemical effect, and readily change the secreting function of the serous membrane.

*Goitre.*—Goitre is occasionally affected most decidedly even when the applications are external. A case of this kind that we have treated at Demilt Dispensary, has decreased in size at least one and a half inches under external galvanization frequently repeated.

*Adenitis.*—Such cases yield with difficulty, require much treatment, and are sometimes as obstinate as malignant tumors.

*Rhinitis* (*Catarrh of the Nose*).—In this troublesome and persistent affection, the effects of local galvanization, both external and internal, are frequently most satisfactory.

CASE XI. *Chronic Catarrh, of Eight Years' Standing. Complete Recovery under Local Galvanization.*—Miss N., aged thirty, directed to us by Dr. Roosa, had suffered for eight years from an aggravated form of the disease. The usual remedies applied by means of the posterior nasal syringe were not effective in relieving her condition. The use of a mild galvanic current, applied externally and directly to the mucous membrane, repeated four times a week for the space of three months, completely and permanently restored the parts to their normal condition.

The possibility of introducing medicines into the system, through the agency of the galvanic current, is a question that has excited considerable interest among some of the German investigators. Fabre-Palaprat and Orioli both claim to have practised this method successfully, the former having experimented with the iodide of potassium, and the latter with corrosive sublimate. Recently, Beer, of Vienna, has experimented in introducing iodine into the body, using small glass cups. After filling one of these cups with a solution of iodide of potassium, and the other with glycerine, a piece of dried bladder or chamois-skin is fastened securely over the mouth of each cup to prevent the escape of the liquid. If, now, we connect the glasses containing the iodide of potassium and the glycerine with the negative and positive poles respectively, and bring the coverings of the cups in contact, the glycerine in the positive cup changes to a violet color, indicating the presence of iodine. If moist flesh be placed between the glasses, the same phenomenon is observed. The rapidity with which this effect is produced depends on the thickness of the flesh. The greater the extent of the tissue through which the current must pass, the slower does the glycerine change in color. If, instead of a single piece of flesh, a number of thin slices, each covered with glycerine, are placed one upon another, and introduced between the cups, the glycerine in the positive cup will as before become colored, while the glycerine on the flesh exhibits no change. Afterward if the flesh is submitted to the direct action of the currect, a dark-blue color appears at the positive pole.

The results obtained by experimenting on the living body were by no means uniform. A patient suffering from ulcer of the knee-joint was subjected to the application, through the leg, of a current with iodine in the circuit for two hours before amputation. No result was obtained.

In another case the result was more satisfactory. The negative glass with a solution of iodide of potassiom was placed on one cheek, while a moist sponge connected with the positive pole was applied to the other cheek. After ten minutes, an examination showed that no iodine was in the saliva. In twelve minutes a slight trace was detected. In fifteen minutes an undoubted trace, and in three-quarters of an hour the saliva changed to a blue color when tested with glycerine.

Beer claims to have benefitted by this method, and after ordinary galvanization had failed, a number of cases of goitre, muscular contraction, and inflammation of joints.—*New York Medical Journal.*

